# The Role of Immune Dysfunction in Parkinson’s Disease Development

**DOI:** 10.3390/ijms242316766

**Published:** 2023-11-26

**Authors:** Davide Cossu, Taku Hatano, Nobutaka Hattori

**Affiliations:** 1 Department of Neurology, Juntendo University, Tokyo 1138431, Japan; 2 Department of Biomedical Sciences, Sassari University, 07100 Sassari, Italy; 3 Neurodegenerative Disorders Collaborative Laboratory, RIKEN Center for Brain Science, Saitama 3510918, Japan

**Keywords:** Parkinson’s disease, immunity, neuroinflammation, mitochondria, dysbiosis, infections

## Abstract

Recent research has unveiled intriguing insights suggesting that the body’s immune system may be implicated in Parkinson’s disease (PD) development. Studies have observed disparities in pro-inflammatory and anti-inflammatory markers between PD patients and healthy individuals. This finding underscores the potential influence of immune system dysfunction in the genesis of this condition. A dysfunctional immune system can serve as a primary catalyst for systemic inflammation in the body, which may contribute to the emergence of various brain disorders. The identification of several genes associated with PD, as well as their connection to neuroinflammation, raises the likelihood of disease susceptibility. Moreover, advancing age and mitochondrial dysfunction can weaken the immune system, potentially implicating them in the onset of the disease, particularly among older individuals. Compromised integrity of the blood–brain barrier could facilitate the immune system’s access to brain tissue. This exposure may lead to encounters with native antigens or infections, potentially triggering an autoimmune response. Furthermore, there is mounting evidence supporting the notion that gut dysbiosis might represent an initial trigger for brain inflammation, ultimately promoting neurodegeneration. In this comprehensive review, we will delve into the numerous hypotheses surrounding the role of both innate and adaptive immunity in PD.

## 1. Introduction

Parkinson’s disease (PD) is a multifaceted, progressive neurodegenerative disorder that impacts not only the central nervous system (CNS) but also peripheral organs [1]. It is believed that the development of PD is the result of a complex interplay between genetic and environmental factors, potentially involving an altered immune system [2]. Numerous clinical studies have revealed notable changes in inflammation markers and immune cell populations in both the peripheral blood and cerebrospinal fluid (CSF) of individuals with PD [3]. Additionally, evidence points to the presence of ongoing and end-stage neuroinflammatory processes within the brains of PD patients [4], suggesting a significant role for neuroinflammation in initiating or worsening the neurodegeneration of the dopaminergic nigrostriatal pathway.

PD is primarily characterized by the degeneration of dopaminergic neurons within the substantia nigra pars compacta [5]. Its distinctive pathological feature involves the presence of intracellular α-synuclein (α-syn) aggregates, commonly known as Lewy bodies [6]. α-syn, in this context, assumes a beta-sheet-rich conformation, facilitating the formation of noxious oligomeric clusters that amass within the nerve fibers of both the central and peripheral nervous systems [7]. These accumulations of α-syn can be expelled from cells and subsequently taken up by adjacent cellular structures like cell bodies, dendrites, or axons [8]. Recent research has hinted at the possibility that α-syn pathology may originate outside the CNS, implying an intricate interaction between brain-resident immune cells and the peripheral immune system [9]. A novel assay system, named immunoprecipitation-based real-time quaking-induced conversion (IP/RT-QuIC), can efficiently detect α-syn seeds in various tissues and blood of PD patients [6]. Histopathological evidence of phosphorylated α-syn has been detected not only in the brain but also in the spinal cord [10], cervical and thoracic ganglia [11], as well as various peripheral organs and the gastrointestinal tract [12]. Concerning the latter, it is conceivable that the gut–brain axis plays a significant role in PD’s pathogenesis, with α-syn potentially generated in the intestinal tract capable of migrating into the bloodstream or even reaching the brain by traversing along the vagus nerve or sympathetic nerves [13]. The fact that people with PD frequently show an increased occurrence of T-cells that identify α-syn peptides strongly implies the participation of autoimmune mechanisms in disease development [14]. A-syn-reactive T-cells are most prevalent shortly after the diagnosis of motor PD and might exist years prior to the identification of motor PD [15].

**Figure 1:**
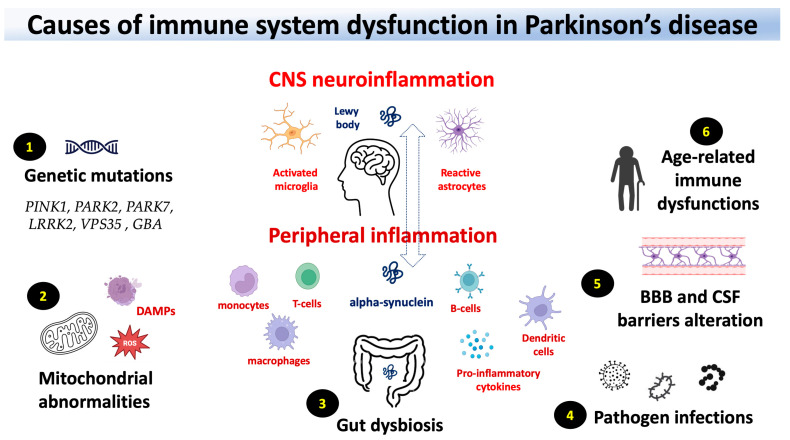
Potential contributors to immune system dysfunction in Parkinson’s Disease (PD). (**1**) Genetic variations associated with an increased risk of PD may also influence immune functions. (**2**) Dysfunction in mitochondria has been linked to both immune system activation and neural degeneration in PD. (**3**) According to the Braak hypothesis, Lewy body pathologies may initiate in the peripheral regions such as the nose and the gut. (**4**) Epidemiological evidence suggests that during the prodromal stage, a combination of factors, including infections, may contribute to the risk of developing PD. (**5**) Increased leakage of blood–brain barrier (BBB) and cerebrospinal fluid barrier (CSFB) in PD patients and animal models points to potential involvement of peripheral immune response and altered drug efficacy in disease progression. (**6**) Age-associated changes in the immune system may increase susceptibility to infection and age-acquired autoimmunity. These factors collectively contribute to peripheral (involving activated innate cells and B/T-cell signaling in the enteric nervous system and blood) and central nervous system inflammation (involving activated microglia and astrocytes).

In this review, we examine the relationship between genes associated with PD and the immune system, the blood–brain barrier changes in PD, and the role of peripheral and CNS innate immunity. Finally, we explore the importance of the microbiota as well as the role of mitochondrial dysfunction in immunity and infections (Figure 1).

## 2. PD Genes Associated with the Function of the Immune System

While most cases of PD are considered idiopathic, it is worth noting that mutations in over 20 genes have been identified, leading to monogenic forms of PD, which account for approximately 5–10% of all PD cases [16]. On the other hand, the vast majority of cases are attributed to polygenic inheritance, where a complex interplay of environmental factors, aging, and genetic predisposition contributes to the development of PD pathology [17].

Some of the genes associated with monogenic PD (Table 1) have potential roles in regulating immune responses, and there are shared genetic variants between PD patients and individuals with autoimmune and inflammatory diseases [18]. For instance, a common non-coding single nucleotide polymorphism known as *rs3129882* in major histocompatibility complex, class II, DR alpha (HLA-DRA), located within the MHC-II locus, has been linked to an increased susceptibility to idiopathic PD [19]. This association may be related to the presentation of pathogenic, immunodominant antigens or a shift towards a more pro-inflammatory CD4 T-cell response, particularly in response to specific environmental exposures like pyrethroid exposure, possibly through genetic or epigenetic mechanisms that influence MHC-II gene expression [19].

Variations in genes related to mitochondrial function, which are a part of the genetic factors contributing to the hereditary component of PD, have been associated with the modulation of both innate and adaptive immune pathways [20]. Proteins encoded by autosomal recessive PD genes, such as E3 ubiquitin ligase PARKIN, PTEN-induced kinase 1 (PINK1), and DJ-1, play roles in mitochondrial pathways [21]. Further supporting the link to mitochondrial dysfunction, evidence emerges from models of autosomal-dominant PD associated with mutations in leucine-rich repeat kinase 2 (LRRK2) and alpha-synuclein (SNCA) [22].

*PARKIN* and *PINK1* genes are vital for maintaining mitochondrial homeostasis and quality control by eliminating damaged mitochondria through a process known as mitophagy [23]. However, recent research, including our own investigations, has revealed intriguing connections between these proteins and mechanisms associated with both innate and adaptive immunity [24]. *PARKIN* and *PINK1* actively suppress the formation of mitochondrial-derived vesicles and the presentation of mitochondrial antigens, identifying them as regulators of the immune system [25]. This underscores the potential involvement of autoimmune processes in the progression of PD. The systemic deletion of *PARK2* and *PINK1* exacerbates experimental autoimmune encephalomyelitis (EAE), an animal model of autoimmune inflammatory diseases of the CNS, in terms of disease severity [24,26]. This age-related exacerbation occurs through the modulation of both peripheral and CNS immune responses. The absence of *PINK1* also alters glial innate immune responses and amplifies inflammation-induced neuron death mediated by nitric oxide [27]. Furthermore, intestinal infection with Gram-negative bacteria triggers PD-like symptoms in *PINK1* knockout mice, leading to the development of cytotoxic mitochondria-specific T-cells in both the periphery and the brain [28]. These findings suggest a fascinating interplay between these PD-related genes and the immune system, shedding light on potential immunological mechanisms underlying the disease’s progression.

*PARK7*, responsible for encoding the DJ-1 protein, is another gene that plays a significant role in autosomal recessive early-onset PD and has garnered attention concerning microglial function and neuroinflammation [29]. When DJ-1 function is impaired or lost, microglial cells exhibit upregulation of pro-inflammatory cytokines while simultaneously downregulating anti-inflammatory pathways [30]. This alteration in microglial behavior may contribute to an increased risk of developing PD by amplifying neuroinflammatory processes within these cells. Interestingly, loss of DJ-1 in PD unexpectedly reduces signs of immunoaging, impacting T-cell compartments in humans and mice [31]. DJ-1 regulates immunoaging through hematopoietic-intrinsic and naïve-CD8-intrinsic mechanisms, presenting a potential target for aging-related diseases [31].

Expression of the autosomal-dominant PD-associated *SNCA* gene, which encodes α-syn, has been observed in various hematopoietic and immune cells [32]. This suggests a potential involvement of immune-mediated mechanisms regulated by α-syn in the pathogenesis of PD. It has been reported that alpha-syn exerts its pathogenic effects through non-cell-autonomous neurotoxic actions and induces inflammatory responses in microglia [33]. These activated microglia, in turn, release neurotoxic agents by activating toll-like receptor 2 (TLR2), which acts as a receptor for neuron-derived α-syn. This cascade ultimately contributes to neurodegeneration [33].

Research has provided evidence of the significant role played by α-syn in the maturation and regulation of T-lymphocytes, as demonstrated in α-syn knockout mice (B6;129X1-Sncatm1Ros1/J) [34]. These mice exhibited an increase in the population of CD4 and CD8 double-negative thymocytes, along with notable decreases in the counts of CD4 single-positive and CD8 single-positive T-cells [34]. Furthermore, α-syn-deficient mice also displayed impaired B-cell lymphopoiesis and deficiencies in IgG production. These findings underscore the importance of α-syn in hematopoiesis, B-cell lymphopoiesis, and the adaptive immune response [35].

The *VPS35* gene, an ortholog of Vacuolar Protein Sorting 35, has been associated with late-onset autosomal dominant familial PD in various studies [36]. This gene appears to play a crucial role in regulating the functions and polarization of microglial cells by influencing the trafficking and recycling of immunomodulating receptors and mediators [36].

The glucocerebrosidase (*GBA*) gene, when mutated, significantly elevates the risk of developing PD by 5–30 times [37]. This gene is expressed in immune cells, including monocytes/macrophages and lymphocytes, and has been linked to an abnormal inflammatory response mediated by these immune cells [38]. Research has shown that non-cell-autonomous mechanisms play a role in the development of *GBA1*-linked PD [39]. This has been demonstrated in mouse astrocytes with heterozygous and homozygous *GBA1 D409V* knock-in mutations. These astrocytes not only exhibited widespread issues with lysosomal structure and function but also displayed significant deficiencies in both basal and TLR4-dependent cytokine production [39]. Notably, inhibiting LRRK2 kinase activity normalized lysosomal function and reduced inflammatory responses, suggesting a functional interplay between glucocerebrosidase and LRRK2 activities in astrocytes [39]. Furthermore, positron emission tomography (PET) scans have detected neuroinflammation in brain regions vulnerable to Lewy pathology in individuals with glucocerebrosidase gene mutations who do not have PD [40]. This finding demonstrates an association with pronounced astrocytic and microglial activation, which can exacerbate neuroinflammatory responses.

Pathogenic mutations within the *LRRK2* gene stand as the prevailing genetic culprits behind PD. Interestingly, *LRRK2* exhibits heightened expression within immune cells such as monocytes, neutrophils, and dendritic cells when compared to its expression in neurons or glial cells [41]. However, intriguing evidence suggests a dynamic interplay between *LRRK2* and α-syn in the context of neuroinflammation-driven PD progression [41]. The inhibition of *LRRK2* can substantially mitigate the neuroinflammatory responses triggered by α-syn binding to TLR2 [42]. This inhibition effectively alleviates neuroinflammation-induced dopaminergic neuronal loss in various experimental models, including mouse glioma cells, primary rat microglia, and human microglia cell lines [42]. Furthermore, studies have demonstrated that LRRK2 kinase inhibition attenuates neuroinflammation, gliosis, and cytotoxicity in murine models receiving intracerebral injections of α-syn preformed fibrils, thereby reinforcing the anti-inflammatory potential of LRRK2 kinase inhibition in preclinical settings [43]. Recent investigations employing a chimeric mouse model revealed that replacing mutant *LRRK2* with the wild-type variant of the protein in T- and B-lymphocytes exerts a marked reduction in lipopolysaccharide (LPS)-mediated inflammation [43]. Additionally, this genetic alteration rescues dopaminergic neuron loss within the substantia nigra pars compacta, underscoring the profound influence of *LRRK2* mutations on the adaptive immune system and its significant role in shaping neuropathological outcomes [44]. Notably, the excessive production of interleukin-6 (IL-6) in the periphery emerges as a pivotal factor in driving LRRK2-mediated neurotoxicity. This suggests that signals originating from the peripheral adaptive immune system act as initiators in a cascade of events culminating in the development of PD pathology. Importantly, the neutralization of excessive peripheral IL-6 production has been demonstrated to prevent the loss of dopaminergic neurons within the substantia nigra pars compacta, highlighting the potential therapeutic significance of targeting these peripheral immune signals in PD [44]. Intriguingly, our ongoing research efforts have unveiled preliminary data indicating that the systemic absence of *LRRK2* may have a preventive or ameliorative effect on the development of active-induced EAE. While these findings remain unpublished, they hint at a broader role for the *LRRK2* gene in immune-related neurological disorders.

Targeting gene-mediated immune dysregulation holds promise as a novel therapeutic avenue for mitigating the progression of PD and related neurodegenerative conditions. To expand this approach from monogenic PD to idiopathic cases, biomarkers are essential for categorizing patients according to their underlying disease etiology. Genetic testing in idiopathic PD patients is a prerequisite for identifying individuals suitable for genotype-driven therapies. The existence of this immunological process could be closely linked to the incapacity of certain individuals with inherited mutations in these genes to efficiently remove abnormal α-syn inclusions from their bodies, thereby resulting in the formation of intracellular Lewy bodies and subsequent neuronal loss and worsening the advancement of the condition.

## 3. Blood–Brain Barrier and Blood–Cerebrospinal Barrier in PD

The blood–brain barrier (BBB), composed of brain endothelial cells, and the blood– CSF barrier (BCSF), formed by the tight junctions between choroid plexus epithelial cells, are two crucial anatomical barriers that constitute the primary interface between the extracellular fluids of the brain and the bloodstream [45]. These barriers serve as the foremost guardians, not only regulating the passage of various circulating substances between brain fluids and blood but also governing interactions between the peripheral immune system and the CNS [45]. The malfunction of the blood–CSF barrier and, particularly the BBB, has been associated with the initiation and progression of numerous neurological disorders, including PD [46]. Several clinical studies, including functional imaging of human patients, analysis of postmortem brain specimens, permeability assessments of drugs used for PD treatment, analysis of albumin/IgG levels in the CSF, and animal models induced with toxins, have identified diverse pathological mechanisms responsible for disrupting the BBB [47]. These mechanisms include the breakdown of intercellular junctions, the accumulation of toxic substances, vascular inflammation, and oxidative stress. Among the factors contributing to BBB disruption, notable attention has been directed toward phenotypic changes in astrocytes and endothelial cells, which, together with pericytes, constitute the neurovascular unit [47].

Mitochondrial oxidative stress within brain endothelial cells emerges as a critical factor in multiple pathological processes responsible for BBB disruption [48]. This oxidative stress harms intercellular junctions, contributes to abnormal cerebral angiogenesis, results in neurovascular decoupling, and actively participates in and exacerbates vascular inflammation [48].

Moreover, the precise role of reactive astrogliosis in the pathogenesis of PD is not fully understood, although it seems to be linked to alterations in BBB permeability [49]. Reactive astrogliosis, often accompanied by neuronal death, can result from various insults to the brain, including infection, inflammatory processes, trauma, α-syn accumulation, and ischemia [50]. Conversely, mutations in the *PARKIN* gene are associated with alterations in astrocytes, suggesting the potential involvement of an astrocyte-related mechanism influencing non-autonomous cell death [51]. Similarly, DJ-1 deficiency can disrupt astrocyte-mediated repair processes through the destabilization of Sox9 and impair the astrogliosis response [52]. Dysfunctional astrocytes were observed in PD genetic animal models. Specifically, in *PARKIN* and *PINK1* knockout mice subjected to active EAE, an animal model of neuroinflammation characterized by disrupted BBB, there was a reduced number of astrocyte cells observed during the later chronic stages of the disease, especially in aged mice [24,26]. These findings indicate that under conditions of neuroinflammation and BBB dysfunction, the absence of these genes exacerbates both peripheral and CNS inflammation, highlighting the significance of the immune system and the crosstalk between the periphery and the CNS in PD.

In a three-dimensional model replicating the BBB, it was noted that astrocytes carrying the *LRRK2 G2019S* mutation displayed a pro-inflammatory profile and induced changes in the morphology and function of brain blood vessels [46]. Further examination of postmortem human brain tissue confirmed that the vascular characteristics observed in the in vitro model closely corresponded to alterations seen in the brains of individuals with sporadic PD [46].

While it remains uncertain whether BBB dysfunction is an early event or a consequence of the primary insult in these diseases, it appears that astrocytes play a pivotal role in connecting the pathological processes occurring in the CNS and the periphery through a compromised BBB.

Regarding the BCSF barrier, our understanding of its involvement in regulating alpha-synuclein levels in the CSF is limited. A study suggested that the choroid plexus can facilitate the transport of α-syn between the blood and the CSF [32375819]. However, another group observed no dysfunction of the BCSF barrier or indications of local IgG synthesis in the early stages of PD [53]. Several therapeutic approaches are being explored to target the BBB and BCSF barrier in PD. Lipid and polymeric-based nanoparticles can be engineered to encapsulate therapeutic agents and navigate the BBB. Surface modifications can enhance their ability to cross the barrier and release the payload in the brain. Some studies have investigated the use of dopamine or levodopa-loaded nanoparticles to deliver neuroprotective agents or anti-inflammatory drugs to the brain in PD [54,55]. Focused ultrasound is a non-invasive technique that uses ultrasound waves to temporarily disrupt the BBB, allowing for enhanced drug delivery. This approach has shown promise in preclinical studies for delivering therapeutic agents to specific brain regions affected by PD [56]. Intranasal administration provides a non-invasive route for drug delivery to the brain. Certain substances can bypass the BBB through the olfactory and trigeminal pathways, reaching the brain directly from the nasal cavity [57]. Studies have explored the intranasal delivery of neuroprotective agents and other drugs for potential PD treatment [58]. Utilizing endogenous transport systems to ferry therapeutic agents across the BBB is an active area of research. This involves designing drugs or drug carriers that can hijack existing transport mechanisms, aiming to enhance the specificity and efficiency of drug delivery while minimizing potential side effects [59]. Peptides that can target specific receptors on the BBB have been investigated for their potential to facilitate drug transport across the barrier. These peptides can be conjugated to therapeutic agents to enhance their ability to cross the BBB [60]. Biologics, including antibodies and other large molecules, are being engineered to improve their ability to cross the BBB by modifying their structure. These biologics may target specific pathways involved in PD pathology [61]. These diverse strategies represent a dynamic field of research with the potential to advance the development of more effective and targeted therapies for PD.

## 4. Peripheral and CNS Immune Responses in PD

The dysregulation of both cellular and humoral immune responses in peripheral tissues has been observed in patients with PD across various age groups, disease durations, and severity of motor or psychiatric symptoms. Research has shown that monocytes derived from the peripheral blood of individuals with PD exhibit heightened phagocytic activity when compared to monocytes from control subjects [62]. Additionally, the treatment of PD patient-derived red blood cell-derived extracellular vesicles has been associated with inflammatory sensitization of human monocytes and an increase in the expression of LRRK2 [63].

Interestingly, the expression of TLRs in peripheral immune cells also appears to be implicated in PD pathogenesis. In women with PD, there is a decrease in TLR4 expression, while the expression of TLR2 seems to correlate with the severity of motor symptoms [64]. Notably, monocytes possess the ability to degrade α-syn through their lysosomal system, and mutations in the *GBA* gene, which impair lysosomal function, are associated with an elevated risk of PD [65]. Furthermore, peripheral monocytes can act as antigen-presenting cells by expressing MHC class I and class II molecules, some of which have been linked to PD [62].

Moreover, distinctions in peripheral immune cell populations, including CD8 T-cells and natural killer (NK) cells in peripheral blood, have been identified between individuals with early-onset PD and those with late-onset PD, defined by a disease duration of less or more than five years, respectively [66]. NK cells, which are recognized as the frontline responders within the immune system, play a pivotal role in promoting immune defense mechanisms through cytotoxicity and the secretion of cytokines. In post-mortem examinations of PD patients, NK cells were found in the substantia nigra [67], and there was an increase in the percentage of NK cells within the CNS parenchyma preceding dopaminergic neuronal degeneration. These findings suggest that NK cells migrate from the periphery into the brain, potentially targeting dysfunctional dopaminergic neurons and contributing to their demise during the progression of PD. The interplay between peripheral and CNS immune responses is a multifaceted aspect of PD pathophysiology. Dysregulated immune responses in the periphery, involving monocytes, TLRs, and specific immune cell populations such as NK cells, can impact the development and progression of PD. Anomalies in the adaptive immune response affecting peripheral blood lymphocytes have been implicated in the pathogenesis of PD. Research has demonstrated notable differences in the immune profiles of PD patients when compared to healthy controls, shedding light on potential immune mechanisms underlying the disease. Specifically, individuals with PD exhibit a significantly reduced proportion of CD3 T-cells in their peripheral blood in comparison to control subjects, leading to an altered CD4 to CD8 T-cell ratio [68]. This observation highlights an abnormality in T-cell populations in PD patients, which is of considerable interest in understanding the disease’s immune aspect.

Furthermore, the peripheral immune profile seen in PD patients deviates from what is typically observed in an older population. Unlike the expected CD8 T-cell replicative senescence associated with normal aging, this phenomenon is notably absent in PD patients [69]. This atypical immune profile suggests that there may be unique immune mechanisms at play in PD, distinct from the immunological changes seen in the aging process. Intriguingly, peripheral CD4 and CD8 T-cells in PD patients have been found to produce Th1/Th2 cytokines in response to α-syn, suggesting the presence of a chronic memory T-cell response in PD, potentially contributing to the ongoing neuroinflammation [14]. Moreover, a recent animal study utilizing an adeno-associated viral model to induce α-syn overexpression in the substantia nigra pars compacta of mice has revealed that α-synuclein overexpression in the mouse midbrain leads to the infiltration of both CD8 and CD4 T-cells into the CNS. Notably, CD4 T-cells play a particularly significant role in mediating α-syn-induced myeloid inflammation and neurodegeneration in this context [70]. Additional preclinical animal models have also indicated dysregulation within the CD8 T-cell compartment. For instance, in PARKIN and PINK1 knockout mice exposed to peripheral inflammation induced by conditions such as EAE or infection with Gram-negative bacteria, a substantial increase in peripheral CD8 T-cell numbers was observed when compared to control animals [24,26,28]. In a recent and sophisticated study analyzing over 700 immune features, an elevated cytotoxic immune profile was identified in early-to-mid-stage idiopathic PD, especially in females [71]. This profile encompassed increased terminally differentiated effector memory (TEMRA) CD8 T-cells, CD8 natural killer T (NKT) cells, and circulating cytotoxic molecules. Selective intensification of co-expression of cytotoxic molecules occurred in CD8 TEMRA and effector memory cells. Regarding the CNS immune responses, studies have identified activated microglia in post-mortem brain tissue from patients diagnosed with PD [72]. Brain PET imaging studies have further revealed the presence of activated microglia, which were observed bilaterally in the midbrain, with a more pronounced presence on the side most affected by the disease [73]. In pathological conditions such as neurodegeneration, microglia, which are the resident macrophages of the CNS, respond promptly to disruptions in brain homeostasis. They migrate to the site of injury and release both pro-inflammatory and anti-inflammatory factors. Microglia also play a crucial role in activating astrocytes [74]. In vitro studies have shown that microglia phagocytose oligomeric α-syn, resulting in the activation of a pro-inflammatory phenotype in these cells. α-syn appears to influence microglial activation through TLRs and other proteins, such as integrin CD11b [75]. Recent research utilizing a seeding/spreading model of α-syn, which involves the injection of α-syn preformed fibrils into the mouse brain, has demonstrated the significant role of microglia in the pathogenesis of PD. Notably, it has been shown that neurodegeneration and microgliosis can occur independently of the formation of α-syn inclusions [76]. It appears that the early stages of PD progression are more likely driven by microglia, activated by oligomeric α-syn [76]. In a genetic mouse model using *PARKIN* knockout mice, neuroinflammation induced by active EAE leads to a robust activation of M1 microglia in the brains of knockout mice compared to wild-type controls [26]. It is worth noting that the autosomal recessive form of PD is characterized by the absence of Lewy bodies [77], suggesting that mitochondrial impairment can also trigger a microglial inflammatory response. Moreover, in the same genetic model, higher numbers of CD8 T-cells were detected in the brains of knockout mice compared to controls. In PD, there is an increased presence of CD8 T-cells in regions of the brain associated with the disease. Furthermore, there is a notable expansion of clonally distinct terminal effector CD8 T-cells within the CSF of individuals with PD [78].

## 5. Gut Microbiota, Infections, and Mitochondrial Dysfunction in PD

Although the origin of Lewy bodies remains unclear, data from human and animal studies have demonstrated the involvement of numerous neurotropic and non-neurotropic bacterial and viral agents in PD [79], indicating the importance of peripheral and CNS inflammation in the onset or progression of the disease.

One of the most relevant theories, the Braak’s hypothesis, suggests that an enteric nervous system lesion triggered by pathogens can promote α-syn aggregation and subsequently spread from the gut to the CNS via the vagus nerve [80]. According to this model, sporadic PD could be caused by a pathogen entering the CNS via the gut and anterior olfactory structures.

Clinical and neuropathological evidence has reported that alterations in intestinal permeability, also known as leaky gut syndrome, may be associated with changes in the composition of the intestinal microbiota and microbial metabolites, and are related to the accumulation of intestinal α-syn [81].

Increased intestinal permeability and translocation of bacteria and inflammatory bacterial products may lead to an overstimulation of the innate immune system, inflammation, and oxidative stress in the gastrointestinal tract, thus initiating the aggregation and accumulation of α-syn in the enteric nervous system [82]. Consequently, overstimulation of the innate immune system in the gut can produce systemic inflammation and neuroinflammation.

The prodromal phase of PD occurs several years before the typical motor symptoms and begins in extranigral structures, including the olfactory bulb or brainstem nuclei [83]. Indeed, hyposmia, an important non-motor symptom of PD that predicts disease progression, is thought to be related to α-syn aggregates in central olfactory structures [84]. It is possible that an infectious agent continues to replicate during this prodromal phase, triggering the immune response that breaks immune tolerance years later.

Age is the main risk factor for the development and progression of PD; is it known that age-related changes in central thymic tolerance can cause a breakdown of self-tolerance [85], with a T-cell compartment in older individuals mounting weak innate and adaptive immune responses to newly encountered pathogens. Indeed, not only has the role of infection become plausible due to Braak’s hypothesis, but cases of PD and parkinsonism have been described following virus infections, including the more recent severe acute respiratory syndrome coronavirus 2 (*SARS-CoV-2*) [86].

Viral and bacterial infections interfere with peripheral tolerance through the induction of CD8 T-cells, whose functionality decreases with age [87]. Animal models have demonstrated age-associated changes in CD8 and DCs in mice lacking mitophagy-related genes [24]. Furthermore, age-related increased oxidative stress and impaired energy production may make neurons vulnerable to toxicity from infectious agents [88].

In addition, inflammation driven by dysfunctional mitochondria can occur in the context of aging, as mitochondria play a critical role during the pro-inflammatory response against pathogenic infections [89], and neuroinflammation-related mitochondrial dysfunction occurs in different neurodegenerative disorders such as PD [90].

Mitochondrial dysfunction is acknowledged as a contributor to both idiopathic and monogenic forms of PD. While it is still debated whether mitochondrial dysfunction is an initial cause or a consequence of PD, dysfunctional lysosomal degradation, and the resulting accumulation of α-syn, are key pathological features [91]. Moreover, the activation of neuroinflammatory processes through the recognition of mitochondrial-derived damage-associated molecular patterns (mtDAMPs) by microglia plays a significant role in this context [92]. Mitochondria play diverse roles in immune responses, influencing metabolic pathways that can modulate immune cell activity, triggering neuroinflammatory responses.

For instance, the absence of mitophagy-related proteins worsens the acute inflammation caused by several stimuli, including bacterial infection and LPS. Intestinal infection with Gram-negative bacteria in *PINK1* knockout mice elicits the establishment of cytotoxic CD8 T-cells in the periphery and the brain, highlighting the relevance of the gut–brain axis in the PD [28].

Swine fever virus infection stimulates PARKIN and PINK1 expression and mitochondrial translocation, leading to mitochondrial fission and increased mitophagy [93]; hepatitis B protein recruits PARKIN to destroy depolarized mitochondria by regulating PINK1 expression [94]; and *SARS-CoV2* impairs intracellular signaling and affects mitochondrial biogenesis [95]. Several intracellular bacteria, such as mycobacteria, can act as virulence factors by modulating mitochondrial physiology for bacterial survival and immune evasion within host cells by inducing macrophage apoptosis via a mitochondrial pathway in macrophages [96].

**Figure 2:**
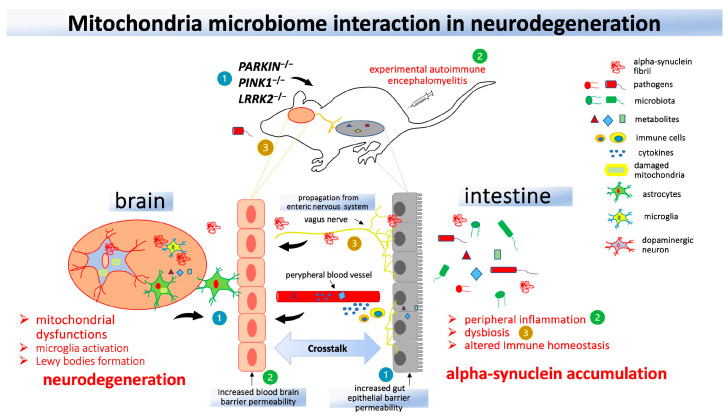
Interaction between mitochondria and the gut in animal models of Parkinson’s Disease (PD). Genetic models of mitochondria knockout animals (**1**) demonstrate an increase in central nervous system neuroinflammation following the induction of experimental autoimmune encephalomyelitis (**2**) or administration of lipopolysaccharide or Gram-negative bacteria (**3**). Mitochondrial dysfunction and peripheral inflammation may contribute to dysbiosis and alpha-synuclein accumulation, which can reach the brain through the vagus nerve and lead to neurodegeneration.

All this evidence shows that deranged mitochondrial activity is involved in inflammation and the dysfunctional response to infection; however, very little is known about the molecular mechanism underlying the change in mitochondrial function in response to pathogens when inflammation occurs in the brain (Figure 2).

Mitochondrial DAMPs, owing to their resemblance to ancient prokaryotes and the similarity they share with pathogen-associated molecular patterns (PAMPs) presented by infectious bacteria, pose a set of challenges to the host immune system. These challenges may result in the immune system responding similarly to mitochondrial DAMPs as it would to a bacterial infection. When mitochondrial quality control pathways fail, it leads to the accumulation of DAMPs, subsequently activating multiple response pathways [97]. These include the TLR9 receptor pathway, the AIM2 and NLRP3 inflammasomes, as well as the cyclic GMP-AMP synthase (cGAS)/stimulator of interferon genes (STING) pathway [97]. This pro-inflammatory stimulus promotes the expression of antimicrobial peptides and virus-targeting interferon (IFN) proteins. It is noteworthy that α-syn exhibits several characteristics akin to antimicrobial peptides [98].

Regarding the association between bacteria and PD, polymorphisms in the *LRRK2* and *PARK7* genes lead to increased susceptibility to PD and confer shared effects on the risk of Crohn’s disease, a human inflammatory bowel disease [99]. These same genetic defects are associated with susceptibility to mycobacteria, including *Mycobacteria tuberculosis* and *Mycobacterium avium* subsp. *paratuberculosis* [100]. *M. tuberculosis* is the causative agent of human tuberculosis and could induce neuroinflammation in astrocytes of PD-related brain regions in an LRRK2-dependent manner [101]. *M. paratuberculosis* is the causative agent of paratuberculosis in ruminants and a suspected causative agent of Crohn’s disease in humans [102]. It has been hypothesized that PD-associated *LRRK2* and *PARK2* defects result in a permissive environment for *M. paratuberculosis* infection and ineffective xerophagy [103], suggesting that, starting as an enteric infection, *M. paratuberculosis*, via the vagus nerve, initiates a pathological process that results in targeted CNS neuroinvasion. On the other hand, studies in animal models have shown that different types of *Bacillus Calmette-Guerin* vaccine can confer non-specific neuroprotection and therapeutic benefits in PD by inducing specific regulatory T (Treg) responses and reducing microglia proliferation and activation [104]. Interestingly, rifampin, an antibiotic used for the treatment of mycobacterial infections, can inhibit microglial inflammation and neurodegeneration induced by α-syn fibrillary aggregates [105].

While there is evidence supporting a genetic component in the susceptibility to PD, most PD cases are considered sporadic, emphasizing the crucial interplay between genetic factors and environmental influences. Genetic predisposition may act as a permissive factor, rendering individuals more vulnerable to environmental insults. The phenomenon of incomplete penetrance, wherein individuals with a genetic predisposition do not always develop the disease, suggests that other factors, such as environmental exposures, including infections or epigenetic modifications, play a significant role in disease manifestation.

Furthermore, whilst each specific disease often exhibits unique characteristics and pathological hallmarks, there might be commonalities in the general mechanisms involving inflammation and neurodegeneration. The hypothesis that something as common as infections can initiate an immune response and inflammation, with chronic inflammation being implicated in neurodegenerative diseases such as Alzheimer’s, PD, and amyotrophic lateral sclerosis, is plausible. This inflammatory response may activate pathways that contribute to neuronal damage, protein misfolding, and aggregation, which are common features across different neurodegenerative disorders.

Delving into the interactions among infections, immune reactions, and PD has the potential to unveil underlying mechanisms that contribute to the initiation and advancement of the disease.

Another crucial aspect in the development of PD is the connection between chaperones, protein homeostasis (the maintenance of proper protein folding and function), and the immune system. Numerous preclinical and clinical studies provide evidence that various molecular chaperones belonging to heat shock protein (HSP) families are either downregulated or dysfunctional in PD [106]. Molecular chaperones not only modulate α-syn but are also implicated in other PD-relevant proteins such as LRRK2 [107], PINK1 [108], PARKIN [109], and DJ-1 [110]. Notably, an increasing body of evidence supports the idea that HSPs directly interact with immune cells [111]. Several HSP families have been identified as prominent antigens in the immune response to various infections [31507418]. The recognition of these conserved antigens not only contributes to protective immunity but may, under certain circumstances, have pathological autoimmune consequences. Moreover, phosphorylated recombinant HSPs have been found to be effective in preserving the integrity of the BBB [112]. All these activities of HSPs could potentially have a mitigating effect on the pro-inflammatory milieu associated with neurological diseases. By reducing neuronal cell death and protein aggregation, HSPs might prevent the initiation of inflammation. Pro-inflammatory mediators released during neuroinflammatory processes may, in turn, modulate the expression levels and activities of HSPs [111]. The alteration in HSP dynamics could indeed have profound implications not only for cellular proteostasis but also for maintaining mitochondrial homeostasis. HSPs localized in the mitochondria, for example, HSP70, play a crucial role in assisting the folding and assembly of mitochondrial proteins, contributing to the overall health and function of the mitochondria within the cell [113]. Understanding the intricate crosstalk between neuroinflammation and HSP functions is pivotal in unraveling the molecular mechanisms contributing to neurodegenerative conditions, offering potential avenues for therapeutic interventions aimed at restoring protein homeostasis in the context of diseases like PD [114].

## 6. Future Perspectives

Understanding the potential role of the immune system in the neurodegenerative processes observed in individuals with PD presents a wide range of opportunities for developing novel treatment approaches. There is significant interest in exploring various strategies, including immunomodulatory drugs and therapies targeting α-syn, to address the complex interplay between neuroinflammation, immunity, and PD pathogenesis.

One avenue of investigation involves examining drugs capable of dampening neuroinflammation, such as non-steroidal anti-inflammatory drugs (NSAIDs), and molecules designed to target specific pro-inflammatory pathways, such as TNF-α inhibitors. These compounds have shown promise in preclinical and clinical studies for their potential to mitigate neuroinflammation in PD. However, their efficacy remains a subject of ongoing debate. The challenge lies in striking the delicate balance between reducing neuroinflammation and preserving essential immune functions. Fully suppressing the immune response could leave PD patients vulnerable to infections, emphasizing the need for careful consideration when employing such treatments.

Another potential therapeutic strategy involves bolstering the function of Tregs. This can be achieved, for instance, through the co-transplantation of autologous Treg cells as a part of cell therapy [115]. This approach holds promise in controlling uncontrolled innate and adaptive immune responses associated with PD.

Additionally, research efforts are focused on developing therapies aimed at reducing the accumulation of α-syn. Vaccines designed to stimulate an immune response against aggregated α-syn, such as α-synuclein mimetic vaccines PD01A and PD03A, are currently in development [116]. These vaccines have the potential to target and clear α-synuclein aggregates, potentially slowing disease progression.

The emerging concept of the gut–brain axis has garnered attention in PD research. Probiotics with neuroprotective properties are undergoing clinical trials [117,118]. These probiotics are designed to modulate the gut microbiome in ways that influence immune responses, potentially impacting the neuroinflammatory processes that contribute to PD pathology. In summary, therapies that can intervene in the early stages of PD may be more effective in slowing or halting the progression of the disease. Given the multifactorial nature of PD, combination therapies that target multiple aspects of the disease may be more effective than single-target approaches. This could involve a combination of neuroprotective strategies, anti-inflammatory agents, and interventions targeting specific genetic factors.

Nevertheless, there are overarching limitations associated with therapeutic approaches addressing key factors in PD. In terms of genetic interventions, PD exhibits genetic heterogeneity, with diverse mutations contributing to its onset. The development of a universally applicable genetic therapy is challenging given this diversity. Ethical concerns linked to gene editing and genetic manipulation, along with considerations of long-term consequences, must be meticulously addressed. A further challenge involves delivering neuroprotective agents across the BBB, which, by restricting the entry of many drugs into the brain, creates difficulties in reaching the affected regions; in some cases, invasive methods may be required to circumvent the BBB, introducing additional risks and complications. When considering the combination of multiple therapeutic agents, potential interactions and side effects require careful consideration. Initiating neuroprotective strategies in the early stages of PD is crucial, yet identifying patients in this phase is challenging, potentially allowing significant neuronal damage to occur by the time symptoms manifest. Additionally, achieving immune system modulation without unintended consequences presents a challenge, as suppressing the immune system may heighten the risk of infections and other complications, requiring a careful balance in treatment. Regarding gut–brain axis interventions, the composition of gut microbiota can vary significantly among individuals. Developing interventions that target this variability while maintaining a balanced gut microbiome presents a challenge. Individual variability in responses to treatments is a noteworthy consideration. Genetic, environmental, and lifestyle factors contribute to variations in individual responses. Therefore, a personalized medicine approach tailored to individual patients based on their specific immune profiles and disease characteristics is a complex but essential endeavor in the pursuit of effective PD treatments.

## Figures and Tables

**Table 1:** Genes associated with PD and the immune system and their role in PD.

Gene Name/Locus	Protein Product	Form of PD/Inheritance	Association with PD and Immune System
*PRKN/PARK2*	Parkin	Juvenile and early-onset PD/autosomal recessive	Inhibit the generation of mitochondrial-derived vesicles and the display of mitochondrial antigens. Function in both peripheral and CNS immunity.
*PINK1/PARK6*	PTEN-induced putative kinase 1	Early-onset PD/autosomal recessive	The loss of PINK1 alters the expression of immunomodulatory genes.
*DJ-1/PARK7*	Protein DJ-1	Early-onset PD/autosomal recessive	DJ-1 loss of function leads to the release of reactive oxygen species, subsequently activating innate immunity and initiating an inflammatory response. DJ-1 regulates immunoaging.
*LRRK2/PARK8*	Leucine-rich repeat kinase 2	Late-onset PD/autosomal dominant	Exhibits robust expression in B-lymphocytes, T-lymphocytes, and peripheral monocytes.
*SNCA/PARK1*	Alpha-synuclein	Early-onset PD/autosomal dominant	Exerts its pathogenic effects through non-cell-autonomous neurotoxic actions and induces inflammatory responses in microglia. It is important for α-syn in hematopoiesis, B-cell lymphopoiesis, and the adaptive immune response.
*VPS35*	Vacuolar Protein Sorting 35	Late-onset PD/autosomal dominant	Plays a pivotal role in modulating the functions and polarization of microglial cells by influencing the trafficking and recycling of receptors and mediators involved in immune regulation.
*GBA*	Beta-Glucocerebrosidase	Early-onset PD/autosomal recessive or dominant (heterozygous carriers)	Mutations result in multi-system inflammation, hyperproliferation of B-lymphocytes, elevated levels of pro-inflammatory cytokines, activation of microglia, and astrogliosis.

## Data Availability

Not applicable.
